# Low-Dose Spironolactone-Pioglitazone-Metformin Normalizes Circulating Fetuin-A Concentrations in Adolescent Girls with Polycystic Ovary Syndrome

**DOI:** 10.1155/2018/4192940

**Published:** 2018-07-19

**Authors:** Marta Díaz, José Miguel Gallego-Escuredo, Abel López-Bermejo, Francis de Zegher, Francesc Villarroya, Lourdes Ibáñez

**Affiliations:** ^1^Endocrinology, Institut Pediàtric Hospital Sant Joan de Déu, University of Barcelona, Esplugues, 08950 Barcelona, Spain; ^2^Centro de Investigación Biomédica en Red de Diabetes y Enfermedades Metabólicas Asociadas (CIBERDEM), ISCIII, Madrid, Spain; ^3^Department of Biochemistry and Molecular Biology, and Institute of Biomedicine, University of Barcelona, 08028 Barcelona, Spain; ^4^Centro de Investigación Biomédica en Red Fisiopatología de la Obesidad y Nutrición (CIBEROBN), ISCIII, Madrid, Spain; ^5^Institut de Recerca Biomèdica de Lleida, Lleida, Spain; ^6^Department of Pediatrics, Dr. Josep Trueta Hospital, Girona Institute for Biomedical Research, 17007 Girona, Spain; ^7^Department of Development and Regeneration, University of Leuven, Leuven, Belgium

## Abstract

**Background:**

Fetuin-A is a glycoprotein produced in the liver and related to metabolic syndrome; fetuin-A secretion is divergently regulated in different pathological conditions. In girls with polycystic ovary syndrome (PCOS), insulin sensitization results in a more favorable endocrine-metabolic outcome than oral contraception; we assessed whether those differences are underscored by changes in circulating fetuin-A.

**Methods:**

Fetuin-A concentration endocrine-metabolic markers and hepatovisceral fat were measured longitudinally in 35 PCOS girls [age, 16 yr; body mass index (BMI), 23 kg/m^2^] randomized to receive either oral contraception [ethinylestradiol-levonorgestrel (*n* = 18)] or a low-dose combination of spironolactone, pioglitazone, and metformin (SPIOMET, *n* = 17) over 12 months. Healthy adolescent girls (age- and BMI-matched) were used as controls (*n* = 25).

**Results:**

Pretreatment fetuin-A serum levels in PCOS girls were lower than those in controls. After 12 months on treatment, fetuin-A raised to control levels only in the SPIOMET subgroup (*P* = 0.009, versus oral contraception); this increase was paralleled by a healthier metabolic profile with less hepatic fat (by MRI); baseline serum fetuin-A as well as the changes over 12 months was inversely related to hepatic adiposity.

**Conclusions:**

A low-dose combination of insulin sensitizers and an antiandrogen—but not oral contraception—normalizes fetuin-A levels in adolescent girls with PCOS. This trial is registered with ISRCTN29234515.

## 1. Introduction

Fetuin-A (*α*2-HS glycoprotein, AHSG) is a glycoprotein produced primarily in the liver and secreted into circulation in high concentrations in humans with fatty liver disease [1]; it binds the insulin receptor and inhibits hepatic and muscle insulin signaling resulting in insulin resistance [2]. In humans, high levels of fetuin-A have been associated with greater risks for type 2 diabetes (T2D) and with features of the metabolic syndrome [3]; paradoxically, increased fetuin-A concentrations prevent vascular calcification and exert a protective role in systemic inflammation, suggesting that fetuin-A secretion can be divergently regulated in different pathological conditions [4].

Polycystic ovary syndrome (PCOS) is the most common cause of hirsutism, acne, and menstrual irregularity in girls and young women and associates to comorbidities in adulthood, including subfertility and T2D. We have previously shown that in adolescent girls with PCOS, oral contraception (OC)—as compared to combined low-dose insulin sensitization plus antiandrogen therapy—is linked to a less favorable endocrine-metabolic profile [5, 6]. To date, the available studies reporting fetuin-A in PCOS have been performed in adult women, include heterogeneous populations, and have a cross-sectional nature [7–9]. Here, we assessed longitudinally whether the divergent effects of oral contraception and low-dose combined insulin sensitization plus antiandrogen therapy in adolescent PCOS are underscored by changes in circulating fetuin-A.

## 2. Subjects and Methods

### 2.1. Study Population and Design

The study population consisted of 35 nonobese adolescent girls with PCOS [mean age, 16 yr; body mass index (BMI), 23 kg/m^2^]; all of them were at least 2 yr beyond menarche. The inclusion criteria were as described [10] (1) hirsutism (score > 8 on Ferriman and Gallwey scale); (2) oligomenorrhea (menstrual intervals > 45 days); and (3) absence of sexual activity throughout the study duration (and thus, no need for contraception). The girls were recruited in the Adolescent Endocrinology Unit of Sant Joan de Déu University Hospital, Barcelona, Spain [10]. Recruitment was biased against overweight/obesity because, in our setting, those girls are primarily referred to the adolescent obesity unit. Exclusion criteria were thyroid dysfunction, anemia, bleeding disorder, hyperprolactinemia, Cushing syndrome, adrenal hyperplasia, liver or kidney dysfunction, and use of drugs affecting gonadal or renal function or carbohydrate or lipid metabolism. Patients were included in a randomized, open-label study comparing the effects of OC with ethinylestradiol-levonorgestrel [EE-LNG; 20 *μ*g of EE plus 100 mg of LNG for 21 of 28 days, placebo for 7 of 28 days; Loette Diario, Pfizer, Madrid, Spain] with those of a low-dose combination of spironolactone 50 mg/d, pioglitazone 7.5 mg/d, and metformin 850 mg/d (SPIOMET). The study was registered as ISRCTN29234515 and lasted for 24 months (12 months on treatment; then 12 months off treatment) [10]. The present report specifically included those girls with complete longitudinal data in whom the remaining serum sample was sufficiently abundant to measure fetuin-A at baseline and after 12 months on treatment (~90% of the initial study population, *n* = 18 and *n* = 17 in the EE-LNG and SPIOMET subgroups, resp.). Twenty-five age- and BMI-matched healthy girls recruited in nearby schools served as controls. All had regular menstrual cycles, and none was hirsute or was on OC or other medications affecting ovarian function or carbohydrate or lipid metabolism.

### 2.2. Clinical and Endocrine-Metabolic Assessments

One investigator (unblinded to treatment) measured weight and height (Harpenden Stadiometer) and scored hirsutism (Ferriman-Gallwey). Systolic and diastolic blood pressures were recorded after a 5-minute rest with the girl supine, using an electronic sphygmomanometer (767 series, Welch Allyn, Spain).

Endocrine-metabolic assessments were performed in the early morning, in the follicular phase (days 3–7) of the cycle or after 2 months of amenorrhea, as described [5]. Briefly, circulating insulin and SHBG were assayed by immunochemiluminescence (IMMULITE 2000, Diagnostic Products, Los Angeles, CA). HOMA-insulin resistance (HOMA-IR) was calculated as [fasting insulin in mU/L] × [fasting glucose in mg/dL]/405. Serum C-reactive protein (CRP) was analyzed by immunochemiluminescence (ARCHITECT i2000SR, Abbott Diagnostics, Abbot Park, IL, USA); intra- and interassay coefficients of variation (CVs) were <10%. HMW adiponectin was assessed by ELISA (R&D Systems, Minneapolis, MN, USA); intra- and interassay CVs were <9%. Circulating fetuin-A was assessed with a specific ELISA (fetuin-A, R&D systems, Minneapolis, MN, USA); the intra- and interassay CVs were 4.2% and 7.4%, respectively.

### 2.3. Abdominal Fat Partitioning

Subcutaneous, visceral, and hepatic fat was assessed by magnetic resonance imaging (MRI) using a multiple-slice MRI 1.5 Tesla scan (Signa LX Echo Speed Plus Excite, General Electric, Milwaukee, WI) [10].

### 2.4. Statistical Analyses and Ethics

Statistical analyses were performed with SPSS 23.0 (SPSS Inc. Chicago, IL). Results are expressed as mean ± SEM. Comparisons within and between groups at each time point were performed using general linear model. Correlation analysis was used to study the associations between fetuin-A levels and auxological and endocrine-metabolic parameters. Two-way analysis of variance (ANOVA) was performed to assess the influence of treatment and time on fetuin-A levels. *P* < 0.05 was considered statistically significant.

The study was conducted after approval by the Institutional Review Board of Sant Joan de Déu University Hospital, after written consent by parents and after assent by each of the participants, including the healthy controls who allowed to obtain indicative values.

## 3. Results

Both treatments reduced androgen excess comparably, but SPIOMET was followed by a more favorable endocrine-metabolic profile, as expected (see Table 1 for differences in selected variables between subgroups).

**Figure 1:**
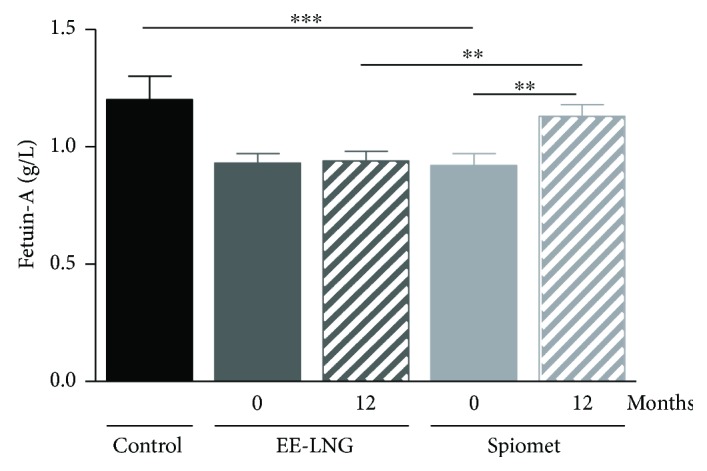
Longitudinal fetuin-A concentrations (mean ± SEM) in adolescent girls with polycystic ovary syndrome who were randomized to receive either an oral contraceptive [ethinylestradiol-levonorgestrel (EE-LNG) *n* = 18] or low-dose spironolactone (50 mg/d), pioglitazone (7.5 mg/d) plus metformin (850 mg/d) (SPIOMET, *n* = 17), for 12 months. Control girls (*n* = 25) matched for age and body mass index were assessed for comparison. ^∗∗∗^*P* < 0.001 for baseline differences between patients and controls; ^∗∗^*P* < 0.01 within patient subgroups for 0 to 12 months and between groups at 12 months.

Pretreatment serum concentrations of fetuin-A in PCOS girls were lower than those in controls. After 12 months on treatment, fetuin-A levels increased only in the SPIOMET subgroup (*P* = 0.009 versus the OC subgroup), reaching control levels (Figure 1, Table 1).

At baseline, circulating fetuin-A correlated negatively with hepatic fat in both controls and PCOS girls (*r* = −0.739; *P* = 0.03 and *r* = −0.446; *P* = 0.006, resp.). After treatment, fetuin-A negatively associated with visceral fat in the SPIOMET subgroup (*r* = −0.583, *P* = 0.004) and with diastolic blood pressure in both the SPIOMET and OC subgroups (*r* = −0.729, *P* = 0.002, and *r* = −0.584, *P* = 0.03, resp.). The change in serum fetuin-A concentrations 0–12 months correlated negatively with diastolic blood pressure (*r* = −0.442; *P* = 0.039), hepatic fat (*r* = −0.647; *P* = 0.002), and C-reactive protein (CRP; *r* = −0 − 617; *P* = 0.003), only in the SPIOMET subgroup. Two-way ANOVA showed that both time and treatment have an effect on fetuin-A levels (*P* = 0.02 and *P* = 0.006, resp.).

## 4. Discussion

Here, we characterize for the first time the longitudinal outcome of circulating fetuin-A concentrations in nonobese adolescents with PCOS and show that fetuin-A levels normalize after treatment with a low-dose combination of insulin sensitizers and an antiandrogen, but not after OC.

Growing evidence supports the relationship between fetuin-A and hepatic fat depots in obesity [11]. Also, strong associations between fetuin-A and insulin resistance have been reported in subjects without diabetes [12]; indeed, fetuin-A promotes insulin resistance through inhibition of insulin receptor tyrosine kinase in hepatocytes and skeletal muscle and activation of Toll-like receptor 4 in response to free fatty acids, stimulating an inflammatory signaling pathway [2, 13]. In contrast, other studies report no associations between fetuin-A and insulin resistance in T2D patients or suggest that fetuin-A is not causally related to diabetes risk [14, 15]. Moreover, the impact of fetuin-A on cardiovascular disease is controversial depending on the presence or absence of diabetes. For example, nondiabetic subjects—but not T2D patients—with higher fetuin-A levels have a lesser risk for cardiovascular disease and related mortality [14]. Besides, fetuin-A would prevent liver and vascular fibrosis through the inhibition of transforming growth factor-*β*1 signaling [16].

Our apparently discordant data could be partially explained bearing in mind that fetuin-A comprises a mixture of circulating isoforms regulating specific biological processes and that the available ELISA assays may differ in their specificity for different glycosylated forms [17]. Moreover, defects of glycosylation/sialylation of fetuin-A leading to protein inactivation have also been reported, indicating that the overall glycosylation status of fetuin-A would also be relevant in the regulation of fetuin-A actions [18].

Fetuin-A also has an anti-inflammatory role, acting as endogenous inhibitor of zinc metalloproteases [19]. This feature could explain the association between fetuin-A concentrations and the change in CRP levels over 12 months in the girls receiving SPIOMET, suggesting that fetuin-A levels within the normal range are required to maintain inflammation homeostasis.

The dual functionality of fetuin-A in diabetes risk, inflammation, and cardiovascular disease raises the question as to whether decreasing fetuin-A concentrations should be customarily recommended [20], especially taking into account the heterogeneity of fetuin-A levels reported in different studies using the same method in similar age groups [21].

The finding of lower levels of fetuin-A in PCOS girls was unexpected and could be derived—at least in part—from the status of low-grade inflammation associated with this entity, since it is known that proinflammatory cytokines and proteins such as CRP—which are increased in PCOS—downregulate fetuin-A expression in the liver [4, 22]. This would also explain the negative association between fetuin-A concentrations and hepatic fat. SPIOMET treatment was followed by a healthier endocrine-metabolic profile, as judged by the reduction of insulin levels and the drop in ectopic fat; in addition, SPIOMET but not OC raised fetuin-A levels towards normal. This finding may be perceived as contradictory, because pioglitazone—but not metformin—has been reported to reduce fetuin-A levels in patients with T2D [23]. However, in those studies, pioglitazone was given in monotherapy, and at doses, at least fourfold higher [23].

The main study limitations include the small sample size and the lack of obese PCOS and obese control girls, precluding to discern the separate effects of obesity and PCOS on fetuin-A levels. The strengths include the longitudinal design, the homogeneous study population, and the assessment of the impact of two interventions with divergent effects on the endocrine-metabolic status and, potentially, on cardiometabolic risk. The cross-sectional nature and population heterogeneity of the so far available studies in PCOS women may explain the discrepancies among populations [7–9].

## 5. Conclusions

In conclusion, we report for the first time that fetuin-A levels are reduced in nonobese girls with PCOS and that a low-dose combination of insulin sensitizers and an antiandrogen—but not an OC—increases towards normal fetuin-A concentrations, together with an improvement of the endocrine-metabolic status. The divergent regulation and thus potential duality of fetuin-A effects in diverse pathological conditions deserve further investigation.

## Figures and Tables

**Table 1:** Data from adolescent girls with polycystic ovary syndrome (PCOS) who were randomized to receive either ethinylestradiol-levonorgestrel (EE-LNG; *n* = 18) or low-dose spironolactone-pioglitazone-metformin (SPIOMET; *n* = 17) for 12 months.

	Controls (*n* = 25)	All PCOS (*n* = 35)	EE-LNG (*n* = 18)			SPIOMET (*n* = 17)		
		Baseline^a^	Baseline	12 months	Δ 0−12 months	Baseline	12 months	Δ 0−12 months
Age (year)	15.6 ± 0.2	15.8 ± 0.2	15.9 ± 0.3	—	—	15.7 ± 0.3	—	—
BMI (kg/m^2^)	22.2 ± 0.5	23.6 ± 0.5	23.9 ± 0.8	24.0 ± 0.8	0.04 ± 0.27	23.1 ± 0.7	23.0 ± 0.7	−0.31 ± 0.20
SBP (mmHg)	112 ± 2	114 ± 1	115 ± 2	112 ± 3	−3.2 ± 2.9	113 ± 2	109 ± 1^b^	−4.9 ± 2.2
DBP (mmHg)	70 ± 1	71 ± 1	72 ± 2	75 ± 2	3.7 ± 2.9	70 ± 1	70 ± 1	−0.5 ± 1.6
AST (UI/L)	17.8 ± 0.7	16.0 ± 0.5	15.7 ± 0.7	16.5 ± 1.1	0.8 ± 0.7	16.3 ± 0.9	17.3 ± 1.0	0.8 ± 0.9
ALT (UI/L)	15.0 ± 1.1	14.2 ± 0.8	15.1 ± 1.3	18.9 ± 2.3^b^	3.9 ± 1.7	13.2 ± 0.6	15.4 ± 1.5	1.7 ± 1.2
GGT (UI/L)	13.9 ± 1.4	12.3 ± 0.4	12.3 ± 0.7	16.4 ± 1.0^d,i^	4.1 ± 0.9	12.2 ± 0.6	10.9 ± 0.4	−1.1 ± 0.6^g^
CRP (nmol/L)	7.5 ± 1.5	15.3 ± 2.3^∗∗^	14.5 ± 2.3	28.5 ± 5.9^b,i^	14 ± 6.6	16.1 ± 4.2	5.5 ± 0.8^c^	−10.6 ± 3.7^f^
N/L ratio	1.6 ± 0.1	1.8 ± 0.1	1.9 ± 0.2	1.8 ± 0.3	−0.1 ± 0.4	1.8 ± 0.1	1.6 ± 0.2	−0.2 ± 0.1
Glucose (mmol/L)	5.1 ± 0.1	4.7 ± 0.1^∗∗∗^	4.7 ± 0.1	4.5 ± 0.1^b^	−0.2 ± 0.1	4.6 ± 0.1	4.3 ± 0.1^c^	−0.3 ± 0.1
Insulin (pmol/L)	56 ± 6	80 ± 7^∗^	92 ± 12	110 ± 18^h^	18 ± 13	70 ± 7	44 ± 7^c^	−27 ± 7^g^
HOMA-IR	1.9 ± 0.2	2.3 ± 0.1	2.5 ± 0.2	2.5 ± 0.3^i^	0.2 ± 0.4	2.1 ± 0.2	1.2 ± 0.2^c^	−0.8 ± 0.2^e^
HDL-C (mmol/L)	1.44 ± 0.04	1.31 ± 0.04^∗^	1.34 ± 0.05	1.35 ± 0.06	0.02 ± 0.05	1.28 ± 0.05	1.40 ± 0.07	0.12 ± 0.06
LDL-C (mmol/L)	2.26 ± 0.11	2.31 ± 0.09	2.31 ± 0.13	2.62 ± 0.19	0.31 ± 0.15	2.30 ± 0.12	2.32 ± 0.10	0.01 ± 0.07
Triglycerides (mmol/L)	0.63 ± 0.05	0.63 ± 0.04	0.60 ± 0.06	0.62 ± 0.04	0.02 ± 0.05	0.66 ± 0.07	0.56 ± 0.05	−0.10 ± 0.05
Testosterone (nmol/L)	0.97 ± 0.05	2.01 ± 0.10^∗∗∗^	2.14 ± 0.19	0.09 ± 0.08^d^	−1.23 ± 0.17	1.89 ± 0.08	0.96 ± 0.08^d^	−0.93 ± 0.09
SHBG (nmol/L)	58 ± 4	29 ± 2^∗∗∗^	30 ± 3	63 ± 7^d,i^	33 ± 6	28 ± 2	32 ± 3	3 ± 3^g^
Fetuin-A (g/L)	1.18 ± 0.05	0.92 ± 0.03^∗∗∗^	0.93 ± 0.04	0.94 ± 0.04^h^	0.01 ± 0.07	0.92 ± 0.05	1.13 ± 0.05^c^	0.23 ± 0.07^e^
Subcutaneus fat (cm^2^)^†^	98 ± 12	150 ± 13^∗^	149 ± 18	142 ± 18	−7.8 ± 9.9	150 ± 19	142 ± 18	−7.8 ± 7.6
Visceral fat (cm^2^)^†^	32 ± 2	46 ± 3^∗^	43 ± 4	42 ± 5	−1.3 ± 3.6	49 ± 5	33 ± 2^b^	−16 ± 5.4^e^
Hepatic fat (%)^†^	12.6 ± 1.4	16.7 ± 1.0^∗^	17.0 ± 1.4	19.8 ± 1.4^i^	2.8 ± 1.4	16.5 ± 1.4	10.1 ± 0.9^c^	−6.4 ± 1.0^g^

Values are mean ± SEM. BMI: body mass index; SBP: systolic blood pressure; DBP: diastolic blood pressure; CRP: c-reactive protein; N/L: neutrophile-to-lymphocyte ratio; HOMA-IR: homeostatic model assessment-insulin resistance; HMW: adiponectin, high molecular weight; TC: total cholesterol; TG, triglycerides; SHBG: sex hormone binding globulin; D4-A: 4-androstenedione; DHEAS: dehydroisoandrosterone sulphate. ^†^By MRI; ^∗^*p* < 0.05, ^∗∗^*p* < 0.01, and ^∗∗∗^*p* < 0.001 between controls and PCOS girls at baseline. ^a^No significant differences between randomized PCOS subgroups at baseline; ^b^*p* < 0.05, ^c^*p* < 0.01, and ^d^*p* < 0.001 within subgroups for 0-to-12-month changes (Δ); ^e^*p* < 0.05, ^f^*p* < 0.01, and ^g^*p* < 0.001 between subgroups for 0-to-12-month changes (Δ); ^h^*p* < 0.01 and ^i^*p* < 0.001 between subgroups at 12 months.
